# Altered asymmetry of functional connectome gradients in major depressive disorder

**DOI:** 10.3389/fnins.2024.1385920

**Published:** 2024-04-30

**Authors:** Yaqian Yang, Yi Zhen, Xin Wang, Longzhao Liu, Yi Zheng, Zhiming Zheng, Hongwei Zheng, Shaoting Tang

**Affiliations:** ^1^School of Mathematical Sciences, Beihang University, Beijing, China; ^2^Key Laboratory of Mathematics, Informatics and Behavioral Semantics, Beihang University, Beijing, China; ^3^Institute of Artificial Intelligence, Beihang University, Beijing, China; ^4^Zhongguancun Laboratory, Beijing, China; ^5^Beijing Advanced Innovation Center for Future Blockchain and Privacy Computing, Beihang University, Beijing, China; ^6^PengCheng Laboratory, Shenzhen, China; ^7^Institute of Medical Artificial Intelligence, Binzhou Medical University, Yantai, China; ^8^State Key Lab of Software Development Environment, Beihang University, Beijing, China; ^9^Beijing Academy of Blockchain and Edge Computing, Beijing, China

**Keywords:** hemispheric asymmetry, functional gradient, major depressive disorder, brain network, resting-state fMRI

## Abstract

**Introduction:**

Major depressive disorder (MDD) is a debilitating disease involving sensory and higher-order cognitive dysfunction. Previous work has shown altered asymmetry in MDD, including abnormal lateralized activation and disrupted hemispheric connectivity. However, it remains unclear whether and how MDD affects functional asymmetries in the context of intrinsic hierarchical organization.

**Methods:**

Here, we evaluate intra- and inter-hemispheric asymmetries of the first three functional gradients, characterizing unimodal-transmodal, visual-somatosensory, and somatomotor/default mode-multiple demand hierarchies, to study MDD-related alterations in overarching system-level architecture.

**Results:**

We find that, relative to the healthy controls, MDD patients exhibit alterations in both primary sensory regions (e.g., visual areas) and transmodal association regions (e.g., default mode areas). We further find these abnormalities are woven in heterogeneous alterations along multiple functional gradients, associated with cognitive terms involving mind, memory, and visual processing. Moreover, through an elastic net model, we observe that both intra- and inter-asymmetric features are predictive of depressive traits measured by BDI-II scores.

**Discussion:**

Altogether, these findings highlight a broad and mixed effect of MDD on functional gradient asymmetry, contributing to a richer understanding of the neurobiological underpinnings in MDD.

## 1 Introduction

Major depressive disorder (MDD) is among the most prevalent psychiatric illnesses worldwide (Bromet et al., 2011; Kyu et al., 2018), characterized by persistent low mood, diminished interests, vegetative symptoms, and increased suicide attempts (Otte et al., 2016). Patients with MDD have been reported to exhibit dysfunction in both sensory perception (e.g., visual and pain perception) and integrative cognitive functions (e.g., memory and social communication; Adler and Gattaz, 1993; Bubl et al., 2010; Fitzgerald, 2013; Kupferberg et al., 2016; Dillon and Pizzagalli, 2018), in parallel with disrupted functional brain connectome in both local and global features (Yang et al., 2021). For example, previous studies have identified alterations in regional functional connectivity, community structures, and network global efficiency (He et al., 2018; Yan et al., 2019; Yang et al., 2021), offering valuable information for the pathophysiology of MDD. However, these pieces of information are fragmented and sometimes inconsistent, leaving the neurobiological mechanisms of cognitive impairments in MDD unclear.

By embedding functional connectomes into a low-dimensional space, functional gradients recently proposed by Margulies et al. (2016) provide an appealing tool to reconcile interactions among distributed brain regions with systematic organizational principles. Regions with similar functional connectivity (FC) profiles are embedded in similar positions along gradient axes, resulting in topographic maps that capture continuous variations of connectivity patterns. In other words, functional gradients depict a continuous spatial arrangement of macroscale brain networks, with variation of functional hierarchy informing how functional connectivity patterns of distinct regions are integrated and segregated (Huntenburg et al., 2018; Bayrak et al., 2019). For example, the principle gradient reflects a macroscale hierarchy where unimodal sensory/motor areas and transmodal default mode areas are situated at two opposite ends, in line with the intrinsic brain geometry that transmodal systems show the greatest geodesic distance from unimodal systems. This unimodal-transmodal gradient hierarchy also corresponds to an increasingly abstract functional spectrum from specialized to integrated information processing. The second gradient captures transitions from visual to somatosensory cortices and the third gradient spans from somatomotor/default mode to multiple demand systems. These gradients characterize regional heterogeneity in a continuous manner, complementary to discrete areas or community identification and mapping (Genon et al., 2021). They also show potential benefits for capturing systematic relationships between distributed cortical regions as well as contextualizing functional patterns of specific regions with global spatial organization (Bernhardt et al., 2022). Moreover, functional gradients have been consistently observed across species (Coletta et al., 2020; Valk et al., 2020; Wan et al., 2022) and have been associated with multi-modal and multi-scale architecture, including variations in gene expression (Burt et al., 2018), microstructure (Paquola et al., 2019b), myeloarchitecture (Huntenburg et al., 2017), and structure-function coupling (Vázquez-Rodŕıguez et al., 2019; Yang et al., 2023), advancing the exploration of brain intrinsic organization. Functional gradients are becoming increasingly common in the literature and have been used to study functional alterations during development and aging (Paquola et al., 2019a; Bethlehem et al., 2020; Larivière et al., 2020) as well as neuropsychiatric disorders (Bayrak et al., 2019; Hong et al., 2019; Dong et al., 2020). In particular, a global compression of the principal gradient and focal deviation of visual, sensorimotor, and default mode network areas have been observed in patients with MDD (Xia et al., 2022), promoting the account for symptoms in MDD that encompass low-level and high-level domains of functioning.

Despite current progress, gradient mapping techniques on MDD considered bilateral systems in brain hemispheres as a whole, and MDD-related alterations in hemispheric patterns remain largely unexplored by using functional gradients. Hemispheric asymmetry is thought to be a critical feature for less redundancy and increased efficiency, affording advantage in parallel and flexible information processing to adapt to sophisticated neurocognitive demands (Hartwigsen et al., 2021). For example, leftward dominance has been associated with language and reasoning capacities whereas rightward dominance is relevant to visuospatial and emotion processing (Demaree et al., 2005; Hagoort and Indefrey, 2014; Chen et al., 2019; Goel, 2019). By taking brain hemispheres into account, previous electroencephalographic, neuroimaging, and behavioral studies have shown considerable evidence for altered patterns of hemispheric asymmetries in depressive disorders. EEG and fMRI findings suggested that depressive disorders displayed disturbed hemispheric activity in frontal and parietal regions, potentially linked to aberrant brain lateralization involved in cognitive and emotional processing (Grimm et al., 2008; Bruder et al., 2017). Studies with dichotic listening and visual hemifield tasks revealed abnormal perceptual asymmetry in depressive subjects (Herrington et al., 2010; Bruder et al., 2012). Connectome analysis pointed to increased normalized local efficiency of the left hemispheric functional networks as well as decreased intra- and inter-hemispheric functional connectivity in MDD (Jiang et al., 2019). Recent evidence from the REST-meta-MDD Project reported that MDD exhibited reduced hemispheric specialization (i.e., increased inter-hemispheric FC relative to intra-hemispheric FC) in broad brain areas, including posterior cingulate cortex, dorsolateral prefrontal cortex, frontal eye fields, and parts of cerebellum and visual cortex (Ding et al., 2021). These hemispheric abnormalities, spanning distributed brain regions and multiple functional systems, are likely to reflect or elicit global deficits of brain organization and lateralization as characterized by the asymmetric disruptions of functional gradients, but empirical research for such alterations is lacking.

Here, we aimed to explore whether, and if so, how MDD influenced the hemispheric asymmetry of brain functional gradients.We employed the diffusion mapping method to evaluate hemispheric functional gradients. Notably, brain operations involve the recruitment of not only separated modules within each hemisphere but also functional cooperation between two hemispheres. Asymmetry of both intra-hemispheric and inter-hemispheric functional connectivity has been reported to be affected in MDD (Van Velzen et al., 2020; Ding et al., 2021). Thereby, we focused on MDD-related alterations in intra-hemispheric and inter-hemispheric gradient asymmetry. The intra- and inter-hemispheric connectivity organizations are suggested to provide complementary information: the former is supposed to reflect hemispheric specialization and corpus callosum inhibition, playing roles in language functions, reasoning, and attention (Gazzaniga, 2000; Wan et al., 2022); the latter is supposed to reflect signal transmission and information integration across both hemispheres, which has been seen for motoric information or rough spatial location information (Gazzaniga, 2000; Wan et al., 2022). These two patterns, mediated by the corpus callosum, are relevant to specialized and integrated information processing (Hartwigsen et al., 2021). With a multivariate approach, we assessed MDD effects on intra-hemispheric and inter-hemispheric gradient asymmetry to provide insights to abnormalities in hemispheric localized organization and cross-hemispheric interplay, separately. Given the overall brain symmetry and the flexible recruitment of hemispheric specialization, we would not expect dramatic differences between MDD and healthy participants. Instead, we hypothesized that there would be small effect sizes distributed across multiple gradient patterns. We finally applied an elastic net model to establish a phenotypic association of asymmetry features. The robustness of results using data after global signal regression (GSR) was also tested.

## 2 Materials and methods

### 2.1 Data acquisition

The MRI data was from the Strategic Research Program for Brain Sciences (SRPBS) Multi-disorder MRI database (Tanaka et al., 2021). All participants in this database provided written informed consent, and all data collection protocols were approved by the institutional review boards of the principal investigators'ss respective institutions. We used MRI data collected from four different sites in the database (UTO, COI, HKH, and KUT). The UTO site was acquired using a 3T GE MR750w scanner, resting-state functional MRI data were collected using the following parameters: TR = 2,500 ms, TE = 30 ms, flip angle = 80°, slice thickness = 3.2 mm, slice gap = 0.8 mm, matrix = 64*64, 40 slices, 240 volumes, in-plane resolution = 3.3*3.3; T1-weighted data were collected using the following parameters: TR = 7.7 ms, TE = 3.1 ms, TI = 400 ms, flip angle = 11°, matrix = 256*256, 1*1*1.2 mm^3^ voxel size. Individuals with MDD were diagnosed based on the Diagnostic and Statistical Manual of Mental Disorders Fourth Edition (DSM-IV) and the Mini-International Neuropsychiatric Interview (MINI). The COI site was acquired using a 3T Siemens Verio. Dot scanner, resting-state functional MRI were collected using the following parameters: TR = 2,500 ms, TE = 30 ms, flip angle = 80°, slice thickness = 3.2 mm, slice gap = 0.8 mm, matrix = 64*64, 40 slices, 240 volumes, in-plane resolution = 3.3*3.3; T1-weighted data were collected using the following parameters: TR = 2,300 ms, TE = 2.98 ms, TI = 900 ms, flip angle = 9°, matrix = 256*256, 1*1*1 mm^3^ voxel size. Individuals with MDD were diagnosed based on the Diagnostic and Statistical Manual of Mental Disorders (DSM) and the Mini-International Neuropsychiatric Interview (MINI). The HKH site was acquired using a 3T Siemens Spectra scanner, resting-state functional MRI data were collected using the following parameters: TR = 2,700 ms, TE = 31 ms, flip angle = 90°, slice thickness = 3 mm, slice gap = 0 mm, matrix = 64*64, 38 slices, 107 volumes, in-plane resolution = 3.0*3.0; T1-weighted data were collected using the following parameters: TR = 1,900 ms, TE = 2.38 ms, TI = 900 ms, flip angle = 10°, matrix = 256*256, 1*1*1 mm^3^ voxel size. Individuals with MDD were diagnosed based on the Diagnostic and Statistical Manual of Mental Disorders (DSM) and the Mini-International Neuropsychiatric Interview (MINI). The KUT site was acquired using a 3T Siemens TimTrio scanner, resting-state function MRI data were collected using the following parameters: TR = 2,500 ms, TE = 30 ms, flip angle = 80°, slice thickness = 3.2 mm, slice gap = 0.8 mm, matrix = 64*64, 40 slices, 240 volumes, in-plane resolution = 3.3125*3.3125; T1-weighted data were collected using the following parameters: TR = 2,000 ms, TE = 3.4 ms, TI = 990 ms, flip angle = 8°, matrix = 240*256, 0.9375*0.9375*1 mm^3^ voxel size. Individuals with MDD were diagnosed based on the Structured Clinical Interview for DSM-IV Axis I Disorders-Patient Edition (SCID). For more details on MRI acquisition see this paper (Tanaka et al., 2021). Due to the extreme imbalance between the number of healthy participants and participants with major depression in three sites (COI, KUT, and UTO), we performed optimal group matching using the R package MatchIt (Stuart et al., 2011). To avoid the possible influence of handedness on the results, we excluded all left-handed participants.

### 2.2 Data preprocessing

Structural and functional data were preprocessed using fMRIPrep 20.2.3 (Esteban et al., 2018a,b; RRID:SCR_016216), which is based on Nipype 1.6.1 (Gorgolewski et al., 2011, 2018; RRID:SCR_002502). In short, each T1w image was corrected for intensity non-uniformity, skull-stripped, brain tissue segmented, and spatial normalization to standard spaces. Each functional data was co-registered to the T1w reference, slice-time corrected, and resampled onto naive space. The preprocessed functional data in naive space was resampled into the standard MNI152NLin6Asym space. The detailed preprocessing description can be found in Supplementary material. The preprocessed data was further denoised by nilearn (Abraham et al., 2014), which included (1) motion scrubbing [Power et al., 2012, 2014; volumes with framewise displacement (FD) > 0.35 or the derivative of root mean square variance over voxels (DVARS) >50, and their neighboring volumes (1 forward and 2 back) were flagged as censored volumes and interpolated using cubic spline], (2) detrending, (3) first-order butterworth filtering (0.01–0.1 Hz), (4) censoring high motion volumes, (5) nuisance regression of 24 head motion parameters (Friston et al., 1996; Satterthwaite et al., 2013), mean cerebrospinal fluid and white matter signals [nuisance regression was performed orthogonally to temporal filtering (Lindquist et al., 2019)], and (6) standardization. Quality control reports of the preprocessing processes generated by fMRIPrep were checked by Y. Y. and Y. Zhen., and functional data with poor T1 image segmentation and inaccurate alignment of functional and anatomical MRI data were excluded. To obtain reliable estimates of functional connectivity, we also excluded participants with <4 min of functional data after motion scrubbing (Parkes et al., 2018). Finally, we used a 400-region homotopic atlas (Yan et al., 2023) to parcellate functional data. In the robustness analysis, we additionally included global signal regression (GSR) and evaluated the influence of GSR on our findings.

### 2.3 Hemispheric functional gradients

For each subject, we reconstructed the functional connectivity (FC) matrix by calculating the Pearson correlation of regional time series, which was subsequently converted to Fisher's *Z*-values. We partitioned FC into four different parts (Supplementary Figure 1): FC within the left hemisphere (LL intra-hemisphere), within the right hemisphere (RR intra-hemisphere), from the left to right hemisphere (LR inter-hemisphere), and from the right to left hemisphere (RL, inter-hemisphere) following previous work (Wan et al., 2022, 2023). Therefore, for each subject, two 200 x 200 intra-hemispheric and two 200 x 200 inter-hemispheric FC matrices (4 matrices per subject) were generated. Intra-hemispheric gradients are obtained from functional connectivity within each hemisphere separately (LL and RR intra-hemispheric matrices) whereas inter-hemispheric gradients are obtained from functional connectivity between two hemispheres (LR and RL inter-hemispheric matrices). For each hemispheric matrix, we estimated the functional gradients using the BrainSpace toolbox (Vos de Wael et al., 2020). Specifically, we thresholded each column of the hemispheric matrix by preserving the top 10% strongest functional connections (Margulies et al., 2016; Hong et al., 2019; Royer et al., 2020). We utilized this thresholded hemispheric matrix to calculate the normalized angle similarity coefficient that captures the similarity of regional FC profiles (Liang et al., 2021). Then, we applied the nonlinear diffusion map embedding (Coifman et al., 2005) to the similarity matrix, obtaining multiple continuous components (i.e., functional gradients) that explain connectome variance in descending order. This algorithm treats the affinity matrix as a graph and estimates a low-dimensional embedding from the high-dimensional connectome matrix. Along these low-dimensional axes (i.e., functional gradients), nodes strongly interconnected by either many suprathreshold connections or few very strong connections are closer together whereas nodes with little or no interconnectivity are farther apart. That is, nodes with similar/dissimilar functional connectivity profiles are embedded closer together/ farther apart along the hierarchy, informing the functional integration and segregation among distinct regions (Huntenburg et al., 2018). The parameter α of this algorithm is set to 0.5 (Margulies et al., 2016). The resultant functional gradients characterize the relevant functional organization of the brain, with the gradient scores associated to brain regions informing their spatial positions within the embedding space. Thus, the differences in gradient scores of brain regions reflect their functional distance along the functional gradient.

To ensure the comparison between functional gradients of the hemispheric matrices as well as the comparability among subjects, we estimated two group-level gradient templates, one for intra-hemisphere and the other for inter-hemisphere. For intra-hemisphere, we average all left and right intra-hemispheric FC matrices (both LL and RR) based on both MDD and healthy controls (HC) and then calculated the group-level gradient templates from this mean intra-hemispheric FC matrix to align the first 10 intra-hemisphere gradient components of each subject via Procrustes rotation. For inter-hemisphere, we calculated the template gradients from a mean inter-hemispheric FC matrix generated by averaging all inter-hemispheric FC matrices (both LR and RL) across all subjects and aligned inter-hemisphere gradient components of each subject to the group-level gradient templates via Procrustes rotation. Procrustes alignment has been widely used to rotate a matrix to maximum similarity with the group-level template without a scaling factor, facilitating comparisons of gradients across different hemispheres and individual subjects (Hong et al., 2019; Dong et al., 2020; Meng et al., 2021; Wan et al., 2022; Xia et al., 2022). Moreover, we also constructed a gradient template exclusively from the HC group and algined individual gradients to this template. Correlations between all-subject aligned gradients and HC-group aligned gradients were high (Pearson *r*_*G*1_*intra*_ = 0.998, *r*_*G*2_*intra*_ = 0.997, *r*_*G*3_*intra*_ = 0.997, *r*_*G*1_*inter*_ = 0.999, *r*_*G*2_*inter*_ = 0.999, *r*_*G*3_*inter*_ = 0.999, all *P* < 0.000).

In agreement with previous studies (Meng et al., 2021; Wan et al., 2022; Xia et al., 2022), we focused on the first three gradient components (G1, G2, and G3) that explained the most of connectome variance (Supplementary Figure 2, which also shows components G4–G10). Each component reflects a well-described functional hierarchical pattern (e.g., G1: unimodal-transmodal gradient; G2: visual-somatosensory gradient; G3: multi-demand gradient). Specifically, G1 explained 19.3 ± 1.8% of the total connectivity variance (MDD, 19.3 ± 1.9%; HC, 19.3 ± 1.7%); G2 explained 14.5 ± 1.3% of connectome variance (MDD, 14.5 ± 1.4%; HC, 14.5 ± 1.3%); G3 explained 11.5 ± 0.8% of connectome variance (MDD, 11.4 ± 0.8%; HC, 11.6 ± 0.8%). There is no statistically significant difference in variance explained in MDD and HC across gradients (two-tailed *t*-test, *P* > 0.1, uncorrected).

### 2.4 Asymmetry index

To assess the hemispheric asymmetry of functional gradients, we introduced an asymmetry index (AI) following (Raemaekers et al., 2018; Liang et al., 2021; Wan et al., 2022). Specifically, for intra-hemispheric asymmetry, AI was calculated as left intra-hemispheric gradient scores minus right intra-hemispheric gradient scores (i.e., LL-RR). A positive AI value indicates leftwards asymmetry, that is, the region in the left hemisphere exhibits a larger gradient score than the homologous region in the right hemisphere. Note that we did not adopt (LL-RR)/(LL+RR), since functional gradient scores contained both positive and negative values and the use of (LL-RR)/(LL+RR) potentially exaggerated the AI values or resulted in discontinuity (Nielsen et al., 2013; Sha et al., 2022). For inter-hemispheric asymmetry, AI was calculated as LR-RL.

### 2.5 MDD-HC comparison

To examine differences in sex distributions between MDD and HC groups, we performed a Chi-square test. To quantify the similarity of spatial maps of hemispheric gradient components, we calculated the Pearson correlation coefficient (*r*) of the gradient scores across regions in the left and right hemispheres.

To assess MDD-related alterations in the gradient asymmetry (AI), we applied multivariate analyzes for both intra-hemispheric and inter-hemispheric patterns. Given that the fMRI data were collected from four acquisition sites, we first corrected for the multi-site effects using a combat harmonization with age, sex, and head motion (mean FD) entered as covariates (Fortin et al., 2018; Yu et al., 2018; Xia et al., 2019, 2022). We then performed MDD-HC comparisons at both region-level and network-level. For region-level analyzes, we assessed the AI scores of each brain region along the first three functional gradients. For network-level analyzes, we aggregated the AI scores of regions by Yeo's seven functional systems (Yeo et al., 2011), which included default mode, limbic, control, salience, dorsal attention, somatomotor, and visual networks. In line with previous work (Wan et al., 2023), the MDD-HC comparisons were conducted using a multivariate linear model whose dependent variable consisted of AI patterns along G1, G2, and G3. Age, sex, and head motion (mean FD) were entered as covariates in the model. We employed this multivariate analysis and used Hotelling's T to identify the shared effects of MDD across the three functional gradients (FDR-corrected *P* < 0.05). We then conducted *post-hoc* analyses to further identify the contribution of each individual gradient to the overall effects, correcting for the number of considered functional gradients (*P* < 0.05/3). The analyses were conducted using the BrainStat toolbox (Larivière et al., 2023).

### 2.6 Meta-analysis

To explore the functional implication and behavioral characterization of brain regions with statistically significant MDD-related alterations, we conducted a Neurosynth meta-analysis (Yarkoni et al., 2011) that associated topic terms with the identified abnormal regions. The meta-analysis was conducted using the python package NiMARE (version 0.1.1; Salo et al., 2023). Specifically, we exploited the Neurosynth's ROI association approach based on the latest version of the Neurosynth database (version-7). We used the vocab-terms annotation approach, which included 3,228 terms for version-7. We only retained the top 25 terms relevant to cognitive behaviors or functions in the results. The analysis was conducted using both hemispheres.

### 2.7 Prediction

We exploited an elastic net model (a linear regression with combined L1 and L2 penalties) to investigate whether the asymmetry scores of the first three functional gradients (3 × 200 = 600 features) could predict depressive traits measured by the Beck Depression Inventory-II (BDI-II). Specifically, we performed a nested cross-validation. We randomly split the individuals into training and test sets (4:1) and repeated 100 times. Multi-site effects were controlled using combat harmonization for training and test sets, separately. For each training set, we applied another 5-fold cross-validation to select the hyperparameters. We varied alpha values from 0.0001 to 1 and selected the one with the minimum mean absolute error (MAE). A predictive model with the optimal alpha was then constructed on all training samples. The model performance was evaluated on the test set by Pearson *r* between the predicted and empirical BDI-II scores. To determine whether our prediction performance exceeded the chance level, a total of 10,000 permutation tests with randomly shuffled BDI-II scores were performed. In the above processes, we performed elastic net with ten L1_ratio values ranging from 0.1 to 1. L1_ratio = 0.5 worked best for intra-hemispheric features and L1_ratio = 0.9 for inter-hemispheric features, and hence, we applied them to our prediction pipeline. The results using other L1_ratio parameters could be found in Supplementary Figure 8.

## 3 Results

We utilized resting-state fMRI data from four sites from the SRPBS Multi-disorder MRI Dataset (unrestricted), including Hiroshima COI (COI, *n* = 97), University of Tokyo Hospital (UTO, *n* = 74), Kyoto University TimTrio (KUT, *n* = 28), and Hiroshima Kajikawa Hospital (HKH, *n* = 42). A total of 109 MDD patients and 132 healthy controls (*n* = 241) were included in our analyzes. All participants were right-handed and there is no statistically significant difference in sex and age between MDD and HC groups (sex: male/female = 56/53 for MDD, male/female = 58/74 for HC, chi-square test, χ^2^ = 1.32, *P* = 0.25; age: 43.29 ± 11.85 for MDD, 42.10 ± 10.71 for HC, *t*-value = 0.13, *P* = 0.89).

### 3.1 Hemispheric asymmetry of functional gradients

**Figure 1:**
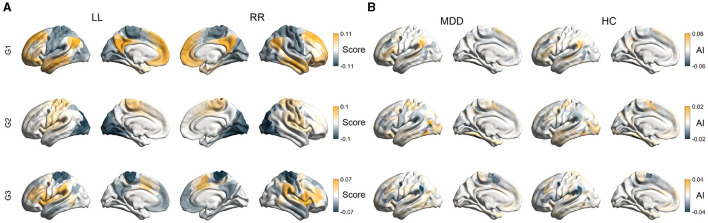
Asymmetry of intra-hemispheric gradients. **(A)** The average intra-hemispheric patterns of the first three gradients across all subjects. LL indicates gradients within the left hemisphere and RR indicates gradients within the right hemisphere. **(B)** Mean asymmetry scores (AI) of the first three gradients (G1, G2, and G3) across subjects in healthy control (HC) and MDD groups.

Through the diffusion map embedding algorithm and the alignment procedure, we generated intra-hemispheric (LL and RR) and inter-hemispheric (LR and RL) maps of the first three functional gradients (G1, G2, and G3) for each subject. Figure 1A illustrated the average intra-hemispheric patterns of the first three gradients across all individuals. Consistent with previous literature (Margulies et al., 2016; Wan et al., 2022, 2023), the principle gradient (G1) of both hemispheres capture a hierarchical organization where unimodal regions (e.g., visual and somatomotor networks) and transmodal regions (e.g., default mode network) were situated at opposite ends. The second gradient component (G2) reveals a hierarchy traversing from visual regions to somatosensory regions. The third gradient component (G3) spans somatomotor/default mode and multiple demand systems. As shown, the spatial patterns of functional gradients were highly similar between the two hemispheres (group-level: *r* = 0.98 for G1, *r* = 0.99 for G2, *r* = 0.97 for G3; individual-level: *r* = 0.86 ± 0.04 for G1, *r* = 0.82 ± 0.07 for G2, *r* = 0.73 ± 0.09 for G3). Results for inter-hemispheric gradients were shown in Supplementary Figure 3A, which exhibited similar patterns to intra-hemispheric gradients.

Despite the overall commonality, there existed some differences/asymmetries between the two hemispheres in both MDD and HC groups. The between-hemispheric differences, indexed by AI scores, were assessed for each subject. The magnitude of AI scores reflects the difference in positions of homologous regions in the left and right hemispheres along functional gradients. Positive AI scores indicate leftwards asymmetry, that is, the region in the left hemisphere exhibits a larger gradient score than the homologous region in the right hemisphere. For instance, regions having positive AI scores in G1 occupy positions closer to the transmodal apex along the principle gradient in the left hemisphere relative to the right hemisphere. Figure 1B presented the group-average AI maps of intra-hemispheric gradient patterns for both MDD and HC participants, and patterns of inter-hemispheric AI maps could be seen in Supplementary Figure 3B. We also reported Cohen's *d* maps for intra- and inter-hemispheric patterns (Supplementary Figures 3C, D), with FDR adjustments for the statistical significance of AI scores (FDR-corrected *P* < 0.05). We found that hemispheric asymmetry was widely present across each of the three gradients. In particular, for the intra-hemispheric gradient G1, the inferior parietal lobule, inferior frontal gyrus, superior frontal gyrus, and superior temporal gyrus exhibited significant leftward asymmetry. In contrast, the middle frontal, lateral occipital lobe and temporal occipital junction, and insular exhibited significant rightward asymmetry.

### 3.2 Network-level analyzes

**Figure 2:**
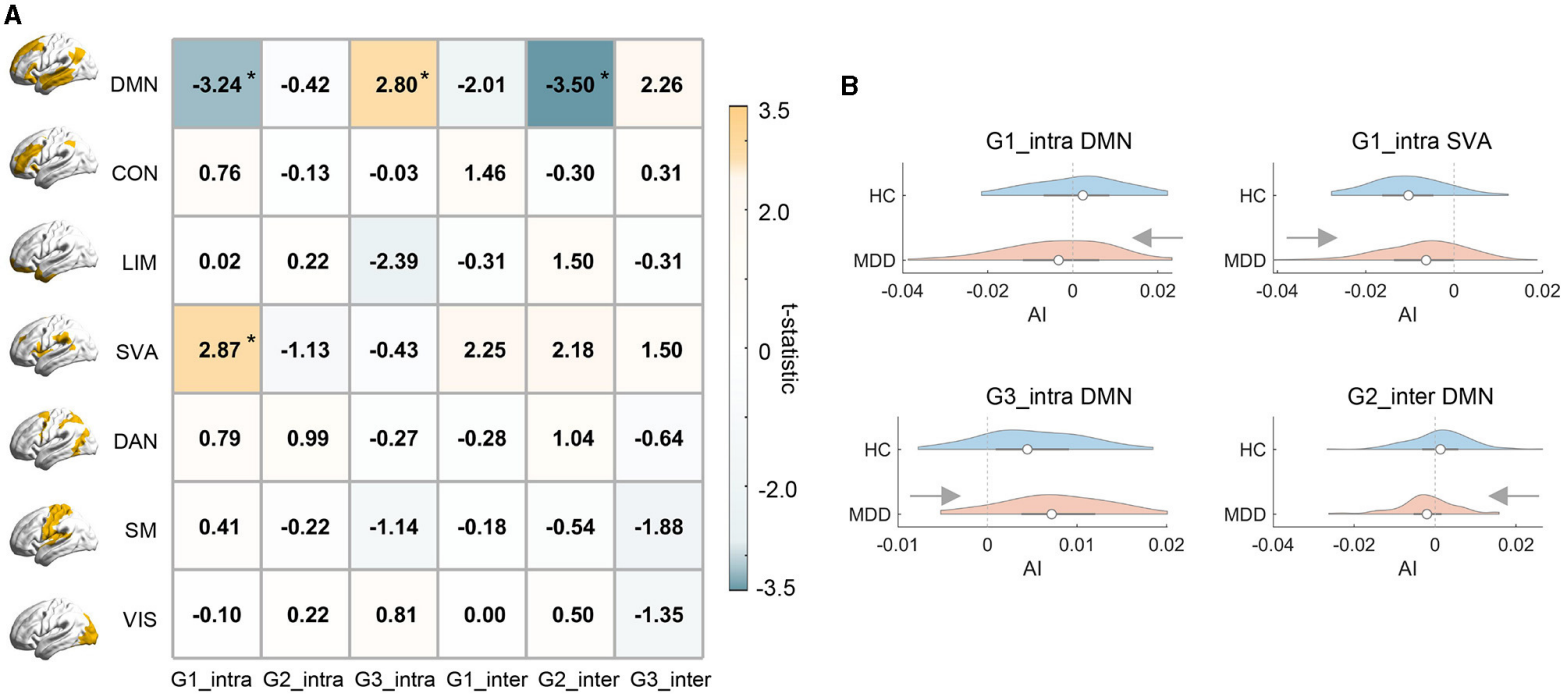
Network-level comparison of asymmetry between MDD and HC groups. **(A)** illustrates the *t*-statistic of the MDD effect on hemispheric asymmetry (AI) for each functional gradient and each RSN. Positive *t*-values indicate larger AI scores of MDD relative to HC and asterisks indicate statistical significance. RSNs: DMN, default mode; CON, control; LIM, limbic; SVA, salience ventral attention; DAN, dorsal attention; SM, somatomotor; VIS, visual networks. **(B)** The violinplot of network-level AI scores across subjects in MDD and HC groups. In each violinplot, the box indicates the interquartile range and the empty circle indicates the median value.

In this section, we attempted to investigate whether MDD altered gradient asymmetries, and if so, whether MDD-related alterations were concentrated in specific functional systems. Broadly, we found that MDD-related alterations in asymmetric organization were concentrated in default mode (DMN) and salience ventral attention (SVA) networks (Figure 2), whose dysfunctions are typically involved with MDD (Kaiser et al., 2015; Mulders et al., 2015). Specifically, for the intra-hemispheric G1, both DMN and SVA exhibited a significant MDD effect on brain lateralization, with DMN showing a shift from leftward to rightward asymmetry (*t* = −3.239, *p* = 0.001) and SVA showing weakened dominance in the right hemisphere (*t* = 2.866, *p* = 0.005). We also found that DMN exhibited increased leftward asymmetry for intra-hemispheric G3 (*t* = 2.799, *p* = 0.006) and a leftward-to-rightward shift for inter-hemispheric G2 (*t* = −3.499, *p* = 0.001). Besides, the alterations in asymmetric organization for SVA came from a decrease in right-hemispheric gradient values while alterations for MDD mainly came from a mixed effect of changes in both left and right hemispheres. Details were reported in the Supplementary Table 1 and results were largely unchanged under global signal regression (GSR; Supplementary Table 2). We also repeated our analyzes with gradient templates contructed exclusively from the HC group. The MDD-HC comparison results were virtually identical (Supplementary Table 3).

### 3.3 Region-level analyzes

**Figure 3:**
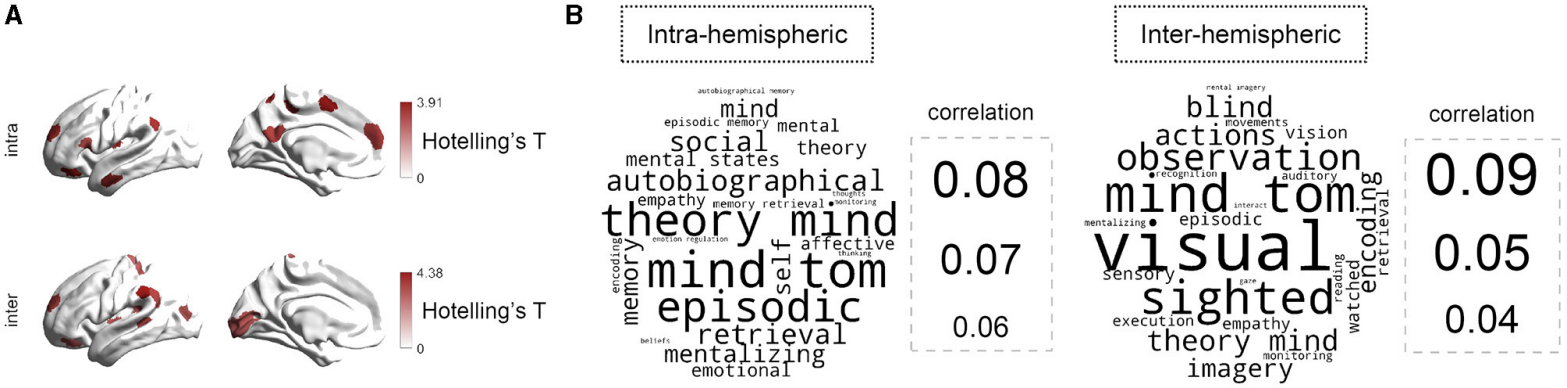
Regional-level comparison of asymmetry between MDD and HC groups. **(A)** Overall MDD-HC differences in hemispheric asymmetry across three gradients (G1, G2, and G3) identified by the multivariate analyzes. The brain maps are colored according to Hotelling's *T*-values (two-sided *P* < 0.05; FDR-corrected). **(B)** Cognitive terms associated with abnormal brain regions. The bigger size of words informs the larger correlation to each topic term.

We next explored the MDD-HC differences of hemispheric gradient asymmetry at the region level. As illustrated in Figure 3A, the multivariate analyzes revealed significant MDD-HC differences in 12 regions for intra-hemispheric patterns and in 13 regions for inter-hemispheric patterns (FDR-corrected *P* < 0.05 for both cases). For intra-hemispheric patterns, these regions were mainly located within prefrontal cortex, lateral temporal cortex, inferior parietal cortex, insula, paracentral lobule, and precuneus/posterior cingulate. For inter-hemispheric patterns, regions with MDD-HC differences included lateral frontal cortex, lateral temporal cortex, insula, inferior parietal cortex, visual cortex, and somatomotor cortex. For single-gradient comparisons, *post-hoc* analyzes showed that MDD-HC differences of asymmetry existed in all three gradients (Supplementary Figure 4). Specifically, for intra-hemispheric G1, MDD-HC differences were mainly located around inferior parietal lobule (IPL_6, *t* = 2.769, *P* = 0.006), posterior cingulate cortex (PCC_3, *t* = −2.882, *P* = 0.004), ventral prefrontal cortex (PFCv_5, *t* = 2.520, *P* = 0.012), temporal cortex (Temp_7, *t* = −2.573, *P* = 0.011), and insula (Ins_1, *t* = −3.621, *P* = 0.000). For intra-hemispheric G2, regions included orbital frontal cortex (OFC_3, *t* = 2.494, *P* = 0.013) and superior parietal lobule (SPL_2, *t* = 3.244, *P* = 0.001). For intra-hemispheric G3, regions included frontal medial cortex (FrMed_2, *t* = −2.713, *P* = 0.007) and somatomotor cortex (SomMot_8, *t* = 3.093, *P* = 0.002). Details were reported in the Supplementary Table 4, which also included results concerning MDD-HC differences in inter-hemispheric asymmetry. We also repeated our analyzes with data after global signal regression (GSR) and the results were roughly consistent (Supplementary Figure 5, Supplementary Table 5). Moreover, we repeated the analyzes with gradient templates contructed exclusively from the HC group. All results were virtually identical (Supplementary Figure 6, Supplementary Table 6).

### 3.4 Cognitive relevance to alterations in MDD

To decode the behavioral relevance of the MDD-altered regions, we applied a NeuroSynth meta-analyzes with 25 cognitive topic terms retained (Margulies et al., 2016). As shown in Figure 3B, for intra-hemispheric asymmetry, regions with significant MDD-HC differences were mainly associated with mind, (episodic and autobiographical) memory, and emotion. For inter-hemispheric patterns, regions with MDD-related alterations were correlated with visualization and mind.

### 3.5 Relevance to phenotypic measures

**Figure 4:**
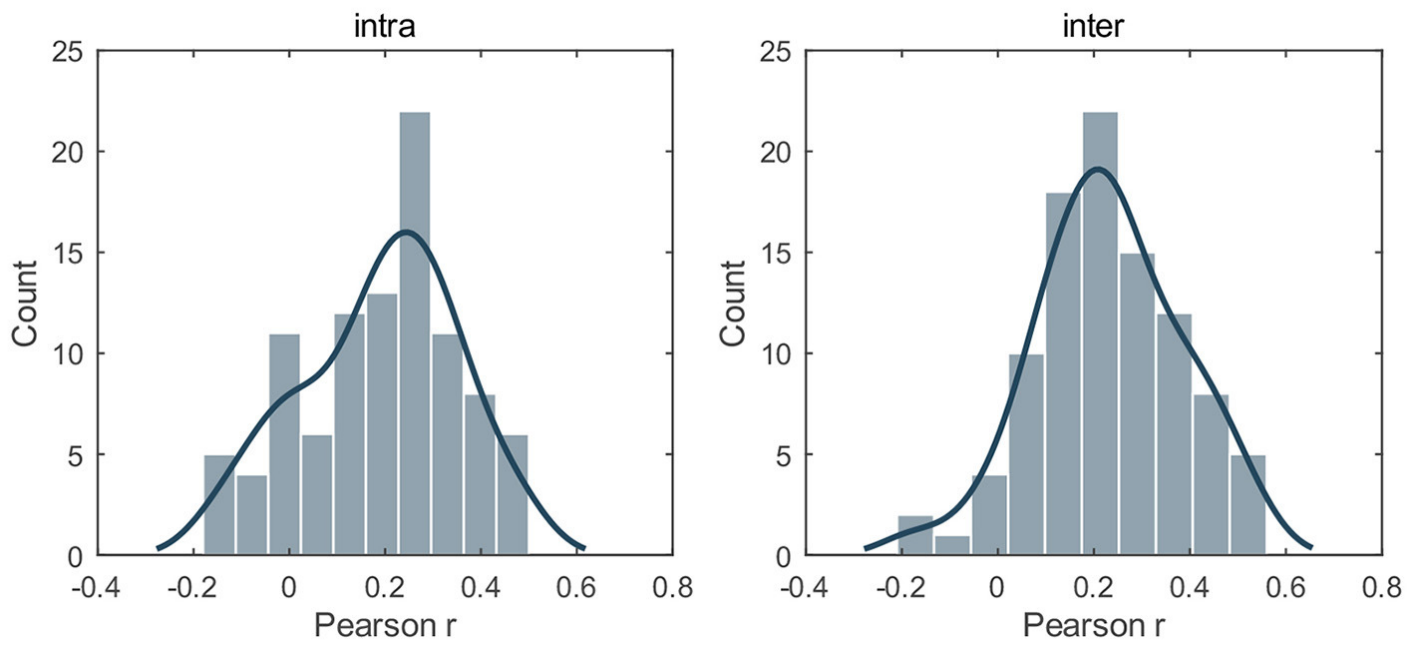
Relation to depression traits. The distribution of Pearson *r* between the predicted and empirical BDI-II scores on test sets across 100 random splits. L1_ratio = 0.5 for intra-hemispheric features and L1_ratio = 0.9 for inter-hemispheric features. For both cases, the prediction accuracy *r* significantly exceeded the chance level (10,000 permutations, *P* < 0.0001). The results using other L1_ratio parameters could be found in Supplementary Figure 8, Supplementary Tables 7, 8.

To examine whether gradient asymmetry could inform depressive traits, we utilized AI scores of the first three gradients to predict BDI-II measures via an elastic net (intra-hemispheric: L1_ratio = 0.5; inter-hemispheric: L1_ratio = 0.9). Supplementary Figure 7 illustrated the frequency of selected features and Figure 4 illustrated the distribution of prediction accuracy *r* on test sets across 100 random splits. As shown, both intra-hemispheric asymmetry and inter-hemispheric asymmetry could significantly predict BDI-II scores, with Pearson *r* being 0.190 ± 0.161 for intra-hemispheric features and being 0.230 ± 0.125 for inter-hemispheric features (empirical *P* < 10^−4^ in both cases). Results using other L1_ratio parameters could be found in Supplementary Figure 8, Supplementary Tables 7, 8.

## 4 Discussion

In the current study, we employed the SRPBS Multi-disorder MRI Dataset, a publicly available, multi-site data of MDD patients and healthy controls (HC) to examine the MDD-related alterations in hemispheric asymmetry of functional gradients. Corresponding to the multi-domain functional deficits in MDD, we found that patients with MDD displayed widespread abnormal asymmetric patterns, including both primary sensory regions (e.g., visual and somatomotor cortices) and higher-order association regions (e.g., prefrontal and default mode areas), implying that MDD may manifest as a consequence of broad disruption of brain efficient lateralized processing. Notably, these abnormalities were distributed across all three functional gradients (G1: unimodal-transmodal gradient; G2: visual-somatosensory gradient; G3: somatomotor/default mode-multiple demand gradient) and for both intra- and inter-hemispheric patterns, which reflect a global and mixed effect of MDD on brain intrinsic organizing principles that depict distinct hierarchical information transitions across the cortex. Besides, both intra- and inter-hemispheric asymmetry features were found to be predictive of depressive traits, indicating MDD-related alterations in both within-hemispheric interaction patterns that underline differentiated processing within each hemisphere and across-hemispheric interaction patterns that support information transfer between two hemispheres. Altogether, our work provides evidence of aberrant asymmetry along functional hierarchies in MDD, advancing our understanding of the pathophysiology of MDD from a new systematic, hemispheric lateralization perspective.

Previous research has revealed connectome gradient dysfunction along fundamental unimodal-transmodal hierarchy in MDD (Xia et al., 2022). Here we extend the findings by assessing hemispheric functional gradients and MDD-HC differences in asymmetry patterns. To our knowledge, this is the first attempt to reveal hemispheric gradient dysfunction in MDD, which is informative for understanding hemispheric lateralized architecture relevant to sensory-cognitive deficits in patients with MDD. Despite the overall similar gradient patterns between the two hemispheres, we found that both MDD and healthy participants exhibited significant left-right differences, with each hemisphere showing relative advantages in particular functional modules. For example, we observed the leftward principal gradient in language-related regions, which has also been consistently observed in previous studies (Liang et al., 2021; Wan et al., 2022, 2023), indicates higher integration of language and transmodal regions in the left hemisphere than in the right hemisphere. In contrast, we found that visual cortices exhibited rightward asymmetry, which may be related to previously reported right dominance of visuospatial function (Fink et al., 2001). Such hemispheric lateralization in functional organization is supposed to facilitate parallel and efficient information processing (Vallortigara and Rogers, 2005; Corballis, 2009), and human advanced neurocognitive operations involve flexible recruitment of brain hemispheric specialized modules to meet a variety of contextual demands (Gazzaniga, 2000; Davis and Cabeza, 2015; Hartwigsen et al., 2021).

By assessing left-right asymmetry of hemispheric functional gradients, we found broad and distributed MDD-HC differences across the brain, spanning from primary sensory regions (e.g., visual and somatomotor cortices) to higher-order association regions (e.g., prefrontal, lateral temporal, and inferior parietal cortices). These identified regions were compatible with the multi-domain functional deficits in MDD and showed somewhat overlap with previous hemispheric studies of depressive disorders. Specifically, the frontal and parietal cortices are mainly involved in cognitive or emotional processing. The approach-withdrawal model has related frontal lateralization to emotional valence (Davidson, 1998; Coan and Allen, 2003) and its expanded model linked parietal asymmetry to emotional arousal (Heller et al., 1995). Despite the mixed results, studies using electrophysiological and neuroimaging measures have reported altered frontal and parietal asymmetry in depressive disorders, which is related to depression and anxiety symptoms (Bruder et al., 2017). Here, abnormal functional gradient asymmetry of frontal and parietal cortices (e.g., prefrontal, lateral frontal, and inferior parietal cortices) was also observed in MDD patients, which provide evidence supporting the involvement of the frontal and parietal asymmetry in the MDD pathology. In addition, depressed individuals have been reported to show poor memory for positive materials and good memory for negative events as well as impaired autobiographical retrieval (Dillon and Pizzagalli, 2018). Our study found aberrant asymmetry of functional gradient organization in part of frontal cortex, parietal cortex, precuneus, and cingulate. These brain regions are involved in attention, episodic memory, and execution functions (Fossati et al., 2002; Fitzgerald et al., 2006; McIntyre et al., 2013). In particular, precuneus has been reported to play a central role in episodic memory retrieval and self-processing (Cavanna and Trimble, 2006). Previous work showed evidence that depressive patients exhibited significantly increased FC among part of frontal cortex, parietal cortex, precuneus, and cingulate with left dorsolateral prefrontal cortex relative to healthy controls (Shen et al., 2015). As functional gradients reflect organizational axes characterizing segregation and integration between distinct regions, our results, as well as the previous findings, suggest that aberrant organization among these brain regions might be implicated in disrupted pattern separation, which potentially suppresses the encoding of positive memories and biases memory retrieval toward negative events (Dillon and Pizzagalli, 2018). Besides, we also observed altered gradient asymmetry of visual cortex and somatomotor cortex in MDD, potentially corresponding to aberrant visual processing and psychomotor retardation in depressive disorders (Buyukdura et al., 2011; Bruder et al., 2017). Consistent with our findings, previous studies have also reported abnormal connectivity asymmetry of visual cortex and decreased regional homogeneity in somatomotor regions (Iwabuchi et al., 2015; Ding et al., 2021). Collectively, our observation of widespread alterations across the cortex could be implicated with affective, cognitive, memory, visual and somatic symptoms in MDD patients. Furthermore, our work provided a potential bridge between these distributed abnormal regions from the perspective of reconfiguration of brain lateralized functional hierarchy, promoting the understanding of how focal alterations and network architecture jointly contribute to the broad pathophysiology of MDD.

Importantly, we found that significant MDD-HC differences were distributed across different functional gradients and that all three gradient features contributed significantly to predicting depressive traits, suggesting that the effects of MDD on brain asymmetry are multidimensional and woven in multiple hierarchical organizations encoding distinct systematic shifts in function. This finding is in line with emerging evidence that hemispheric asymmetry is more complex than could be captured by a single dimension (Liu et al., 2009; Badzakova-Trajkov et al., 2016; Häberling et al., 2016; Corballis, 2019). Previous work has observed abnormalities of hemispheric specialization in DMN (Jiang et al., 2019; Ding et al., 2021). Our results extended this knowledge that MDD-related alterations in DMN manifest as a mixed reconfiguration composed of several independent hemispheric hierarchies (e.g., G1_intra, G3_intra, and G2_inter). Given the critical role of DMN in information transmission and functional integration (Vatansever et al., 2015; Wens et al., 2019; Lanzoni et al., 2020), abnormalities in DMN may induce extensive disruption of polysynaptic signaling along many key communication pathways and ultimately influence different global functional hierarchical patterns. Our observation of multi-dimensional alterations, combined with previously reported altered functional connectivity and functional activation (Yan et al., 2019; Scalabrini et al., 2020), collectively confirmed that aberrant hemispheric specialization in DMN plays an important role in MDD. Furthermore, we found that MDD elicited multiple specialized alterations in DMN across different functional gradients (e.g., left-to-right shift for G1_intra, increased leftward for G3_intra, and left-to-right shift for G2_inter), which may provide an alternative account for inconsistent results of previous work. MDD is likely a mixed consequence of heterogenous lateralized alterations along distinct axes of hierarchy; different analysis strategies and sample populations may bias the observation in favor of a specific pattern of disrupted hemispheric asymmetry. Thus, future investigations of the heterogeneity and complexity of MDD could be extended to multiple dimensions to cover various and complementary information on the underlying neurobiological mechanisms. In addition, we found that both intra-hemispheric and inter-hemispheric asymmetry features were affected by MDD and predictive of depressive traits, implying that MDD-related alterations involve not only hemispheric localized organization that underlies separated operations, but also cross-hemispheric information transfer that mediates bilateral coordination. This is in line with previous work suggesting MDD-related decreases in both intra-hemispheric and inter-hemispheric functional connectivity (Jiang et al., 2019). Moreover, despite the overall spatial similarity, focal alterations of intra- and inter-hemispheric asymmetry exhibited subtle differences in brain regions, which may be due to discrepancy between intra- and inter-hemispheric patterns in terms of their communication characteristics and functional implications. That is, intra-hemispheric organization may reflect localized processing and hemispheric specialization whereas inter-hemispheric organization may reflect information transmission across both hemispheres (Gazzaniga, 2000; Hartwigsen et al., 2021). These two patterns are dissociable from but also intertwined with each other in a flexible manner to conserve a balance of diverse domain-specific and domain-general processes (Genç et al., 2011; Westerhausen et al., 2011; Davis and Cabeza, 2015). The alterations of intra- and inter-hemispheric gradient asymmetry observed here may reflect a reconfiguration of this balance in MDD patients, which deserves further investigation in future work.

There are a few limitations in our study. First, our findings were made by the SRPBS Multi-disorder MRI Dataset where fMRI data were collected from four acquisition sites. Although the Combat harmonization was conducted to correct for the multi-site effects, the current findings need to be validated by large homogenized data collection from one site. Meanwhile, we constrained our analyzes to right-handed adults; future work could include left-handed participants and children to explore the influence of handedness and development. In addition, we only included functional data in our analyzes; further investigation could incorporate multimodal data (e.g., gene expression, cytoarchitecture, myelination, and structural/functional connectivity) to enrich our understanding of MDD pathology.

## Data availability statement

The original contributions presented in the study are included in the article/Supplementary material, further inquiries can be directed to the corresponding authors.

## Ethics statement

The studies involving humans were approved by the University of Tokyo Faculty of Medicine, Hiroshima University, and Kyoto University. The studies were conducted in accordance with the local legislation and institutional requirements. The participants provided their written informed consent to participate in this study.

## Author contributions

YY: Investigation, Methodology, Visualization, Writing – original draft, Writing – review & editing. YZhen: Investigation, Methodology, Visualization, Writing – review & editing. XW: Investigation, Methodology, Writing – review & editing. LL: Investigation, Methodology, Writing – review & editing. YZheng: Investigation, Methodology, Writing – review & editing. ZZ: Investigation, Resources, Supervision, Writing – review & editing. HZ: Investigation, Writing – review & editing. ST: Funding acquisition, Investigation, Writing – review & editing.
