# Supplementary material for: Altered asymmetry of functional connectome gradients in major depressive disorder
Source: Front Neurosci. 2024 Apr 30;18:1385920. doi: 10.3389/fnins.2024.1385920 (PMC11092381; doi:10.3389/fnins.2024.1385920)
Supplement: Supplementary file 2 [file Data_Sheet_2.pdf]

Supplementary Material

1 DATA PREPROCESSING

Results included in this manuscript come from preprocessing performed using *fMRIPrep* 20.2.3 ((Esteban et al., 2018b,a); RRID:SCR_016216), which is based on *Nipype* 1.6.1 ((Gorgolewski et al., 2011, 2018); RRID:SCR_002502).

Anatomical data preprocessing

A total of 1 T1-weighted (T1w) images were found within the input BIDS dataset. The T1-weighted (T1w) image was corrected for intensity non-uniformity (INU) with N4BiasFieldCorrection (Tustison et al., 2010), distributed with ANTs 2.3.3 (Avants et al., 2008, RRID: SCR_004757), and used as T1w-reference throughout the workflow. The T1w-reference was then skull-stripped with a *Nipype* implementation of the antsBrainExtraction.sh workflow (from ANTs), using OASIS30ANTs as target template. Brain tissue segmentation of cerebrospinal fluid (CSF), white-matter (WM), and gray-matter (GM) was performed on the brain-extracted T1w using fast [FSL 5.0.9, RRID:SCR_002823](Zhang et al., 2001). Volume-based spatial normalization to two standard spaces (MNI152NLin6Asym, MNI152NLin2009cAsym) was performed through nonlinear registration with antsRegistration (ANTs 2.3.3), using brain-extracted versions of both T1w reference and the T1w template. The following templates were selected for spatial normalization: *FSL's MNI ICBM 152 non-linear 6th Generation Asymmetric Average Brain Stereotaxic Registration Model* [(Evans et al., 2012), RRID:SCR_002823; TemplateFlow ID: MNI152NLin6Asym], *ICBM 152 Nonlinear Asymmetrical template version 2009c* [(Fonov et al., 2009), RRID:SCR_008796; TemplateFlow ID: MNI152NLin2009cAsym],

Functional data preprocessing

For each of the 1 BOLD runs found per subject (across all tasks and sessions), the following preprocessing was performed. First, a reference volume and its skull-stripped version were generated using a custom methodology of *fMRIPrep*. Susceptibility distortion correction (SDC) was omitted. The BOLD reference was then co-registered to the T1w reference using flirt (FSL 5.0.9, (Jenkinson and Smith, 2001)) with the boundary-based registration (Greve and Fischl, 2009) cost-function. Co-registration was configured with nine degrees of freedom to account for distortions remaining in the BOLD reference. Head-motion parameters with respect to the BOLD reference (transformation matrices, and six corresponding rotation and translation parameters) are estimated before any spatiotemporal filtering using mcflirt (FSL 5.0.9, (Jenkinson et al., 2002)). BOLD runs were slice-time corrected using 3dTshift from AFNI 20160207 (Cox and Hyde, 1997, RRID:SCR_005927). The BOLD time-series (including slice-timing correction when applied) were resampled onto their original, native space by applying the transforms to correct for head-motion. These resampled BOLD time-series will be referred to as *preprocessed BOLD in original space*, or just *preprocessed BOLD*. The BOLD time-series were resampled into standard space, generating a *preprocessed BOLD run in MNI152NLin6Asym space*. First, a reference volume and its skull-stripped version were generated using a custom methodology of *fMRIPrep*. Several confounding time-series were calculated based on the *preprocessed BOLD*: framewise displacement (FD), DVARS and three region-wise global signals. FD was computed using two formulations following Power (absolute sum of relative motions, (Power et al., 2014)) and Jenkinson (relative root mean square displacement between affines, (Jenkinson et al., 2002)). FD and DVARS are calculated for each functional run, both using their implementations in

Nipype (following the definitions by (Power et al., 2014)). The three global signals are extracted within the CSF, the WM, and the whole-brain masks. Additionally, a set of physiological regressors were extracted to allow for component-based noise correction (*CompCor*,(Behzadi et al., 2007)). Principal components are estimated after high-pass filtering the *preprocessed BOLD* time-series (using a discrete cosine filter with 128s cut-off) for the two *CompCor* variants: temporal (tCompCor) and anatomical (aCompCor). tCompCor components are then calculated from the top 2% variable voxels within the brain mask. For aCompCor, three probabilistic masks (CSF, WM and combined CSF+WM) are generated in anatomical space. The implementation differs from that of Behzadi et al. in that instead of eroding the masks by 2 pixels on BOLD space, the aCompCor masks are subtracted a mask of pixels that likely contain a volume fraction of GM. This mask is obtained by thresholding the corresponding partial volume map at 0.05, and it ensures components are not extracted from voxels containing a minimal fraction of GM. Finally, these masks are resampled into BOLD space and binarized by thresholding at 0.99 (as in the original implementation). Components are also calculated separately within the WM and CSF masks. For each CompCor decomposition, the k components with the largest singular values are retained, such that the retained components' time series are sufficient to explain 50 percent of variance across the nuisance mask (CSF, WM, combined, or temporal). The remaining components are dropped from consideration. The head-motion estimates calculated in the correction step were also placed within the corresponding confounds file. The confound time series derived from head motion estimates and global signals were expanded with the inclusion of temporal derivatives and quadratic terms for each (Satterthwaite et al., 2013). Frames that exceeded a threshold of 0.5 mm FD or 1.5 standardised DVARS were annotated as motion outliers. All resamplings can be performed with *a single interpolation step* by composing all the pertinent transformations (i.e. head-motion transform matrices, susceptibility distortion correction when available, and co-registrations to anatomical and output spaces). Gridded (volumetric) resamplings were performed using antsApplyTransforms (ANTs), configured with Lanczos interpolation to minimize the smoothing effects of other kernels (Lanczos, 1964). Non-gridded (surface) resamplings were performed using mri_vol2surf (FreeSurfer).

Many internal operations of *fMRIPrep* use *Nilearn* 0.6.2 [RRID:SCR_001362](Abraham et al., 2014), mostly within the functional processing workflow. For more details of the pipeline, see the section corresponding to workflows in *fMRIPrep*'s documentation.

Copyright Waiver

The above boilerplate text was automatically generated by fMRIPrep with the express intention that users should copy and paste this text into their manuscripts *unchanged*. It is released under the CC0 license.

SUPPLEMENTARY REFERENCES

Abraham, A., Pedregosa, F., Eickenberg, M., Gervais, P., Mueller, A., Kossaifi, J., et al. (2014). EnglishMachine learning for neuroimaging with scikit-learn. *Frontiers in Neuroinformatics* 8. doi:10.3389/fninf.2014.00014

Avants, B., Epstein, C., Grossman, M., and Gee, J. (2008). Symmetric diffeomorphic image registration with cross-correlation: Evaluating automated labeling of elderly and neurodegenerative brain. *Medical Image Analysis* 12, 26–41. doi:10.1016/j.media.2007.06.004

Behzadi, Y., Restom, K., Liau, J., and Liu, T. T. (2007). A component based noise correction method (CompCor) for BOLD and perfusion based fmri. *NeuroImage* 37, 90–101. doi:10.1016/j.neuroimage.2007.04.042

Cox, R. W. and Hyde, J. S. (1997). Software tools for analysis and visualization of fmri data. *NMR in Biomedicine* 10, 171–178. doi:10.1002/(SICI)1099-1492(199706/08)10:4/5⟨171::AID-NBM453⟩3.0.CO;2-L

Esteban, O., Blair, R., Markiewicz, C. J., Berleant, S. L., Moodie, C., Ma, F., et al. (2018a). fmriprep. *Software* doi:10.5281/zenodo.852659

Esteban, O., Markiewicz, C., Blair, R. W., Moodie, C., Isik, A. I., Erramuzpe Aliaga, A., et al. (2018b). fMRIPrep: a robust preprocessing pipeline for functional MRI. *Nature Methods* doi:10.1038/s41592-018-0235-4

Evans, A., Janke, A., Collins, D., and Baillet, S. (2012). Brain templates and atlases. *NeuroImage* 62, 911–922. doi:10.1016/j.neuroimage.2012.01.024

Fonov, V., Evans, A., McKinstry, R., Almli, C., and Collins, D. (2009). Unbiased nonlinear average age-appropriate brain templates from birth to adulthood. *NeuroImage* 47, Supplement 1, S102. doi:10.1016/S1053-8119(09)70884-5

Gorgolewski, K., Burns, C. D., Madison, C., Clark, D., Halchenko, Y. O., Waskom, M. L., et al. (2011). Nipype: a flexible, lightweight and extensible neuroimaging data processing framework in python. *Frontiers in Neuroinformatics* 5, 13. doi:10.3389/fninf.2011.00013

Gorgolewski, K. J., Esteban, O., Markiewicz, C. J., Ziegler, E., Ellis, D. G., Notter, M. P., et al. (2018). Nipype. *Software* doi:10.5281/zenodo.596855

Greve, D. N. and Fischl, B. (2009). Accurate and robust brain image alignment using boundary-based registration. *NeuroImage* 48, 63–72. doi:10.1016/j.neuroimage.2009.06.060

Jenkinson, M., Bannister, P., Brady, M., and Smith, S. (2002). Improved optimization for the robust and accurate linear registration and motion correction of brain images. *NeuroImage* 17, 825–841. doi:10.1006/nimg.2002.1132

Jenkinson, M. and Smith, S. (2001). A global optimisation method for robust affine registration of brain images. *Medical Image Analysis* 5, 143–156. doi:10.1016/S1361-8415(01)00036-6

Lanczos, C. (1964). Evaluation of noisy data. *Journal of the Society for Industrial and Applied Mathematics Series B Numerical Analysis* 1, 76–85. doi:10.1137/0701007

Power, J. D., Mitra, A., Laumann, T. O., Snyder, A. Z., Schlaggar, B. L., and Petersen, S. E. (2014). Methods to detect, characterize, and remove motion artifact in resting state fmri. *NeuroImage* 84, 320–341. doi:10.1016/j.neuroimage.2013.08.048

Satterthwaite, T. D., Elliott, M. A., Gerraty, R. T., Ruparel, K., Loughead, J., Calkins, M. E., et al. (2013). An improved framework for confound regression and filtering for control of motion artifact in the preprocessing of resting-state functional connectivity data. *NeuroImage* 64, 240–256. doi:10.1016/j.neuroimage.2012.08.052

Tustison, N. J., Avants, B. B., Cook, P. A., Zheng, Y., Egan, A., Yushkevich, P. A., et al. (2010). N4itk: Improved n3 bias correction. *IEEE Transactions on Medical Imaging* 29, 1310–1320. doi:10.1109/TMI.2010.2046908

Zhang, Y., Brady, M., and Smith, S. (2001). Segmentation of brain MR images through a hidden markov random field model and the expectation-maximization algorithm. *IEEE Transactions on Medical Imaging* 20, 45–57. doi:10.1109/42.906424

2 SUPPLEMENTARY FIGURES

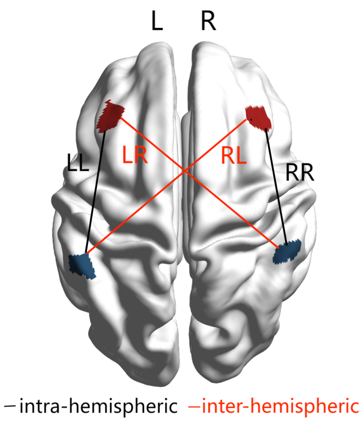

Figure S1. Intra-hemispheric and inter-hemispheric FC patterns. That is, FC within the left hemisphere (LL intra-hemisphere), FC within the right hemisphere (RR intra-hemisphere), FC from the left to right hemisphere (LR inter-hemisphere), and FC from the right to left hemisphere (RL, inter-hemisphere).

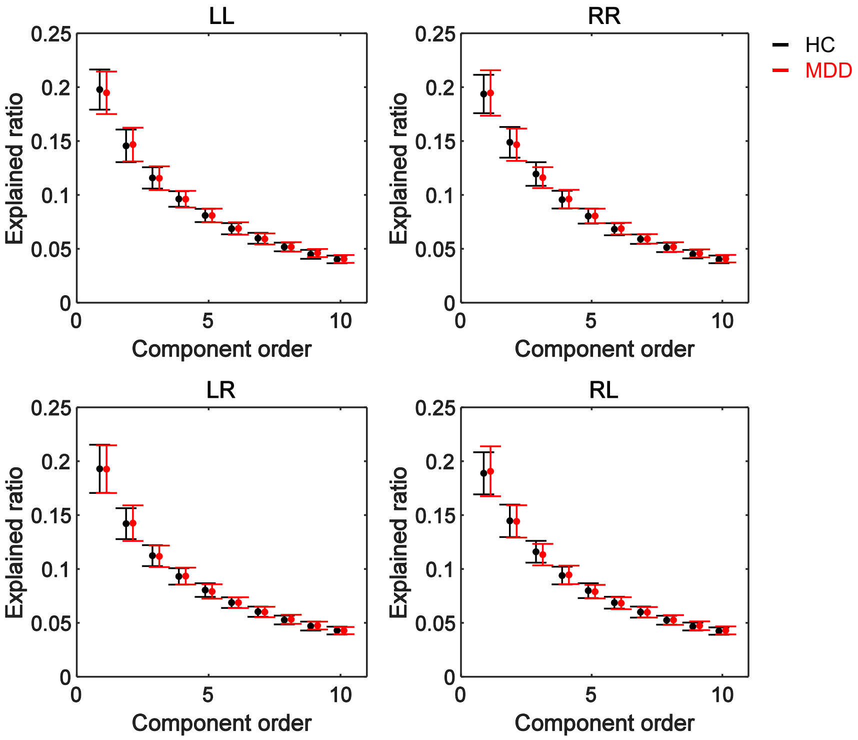

Figure S2. Connectome variance explained by the first 10 gradient components. Healthy controls (HC) are shown in black and MDD patients are shown in red. Dots indicate the mean values and error bars indicate one standard deviation. There were no statistically significant differences in variance explained across the components (two-tailed Student's t-test; $p > 0.1$ uncorrected).

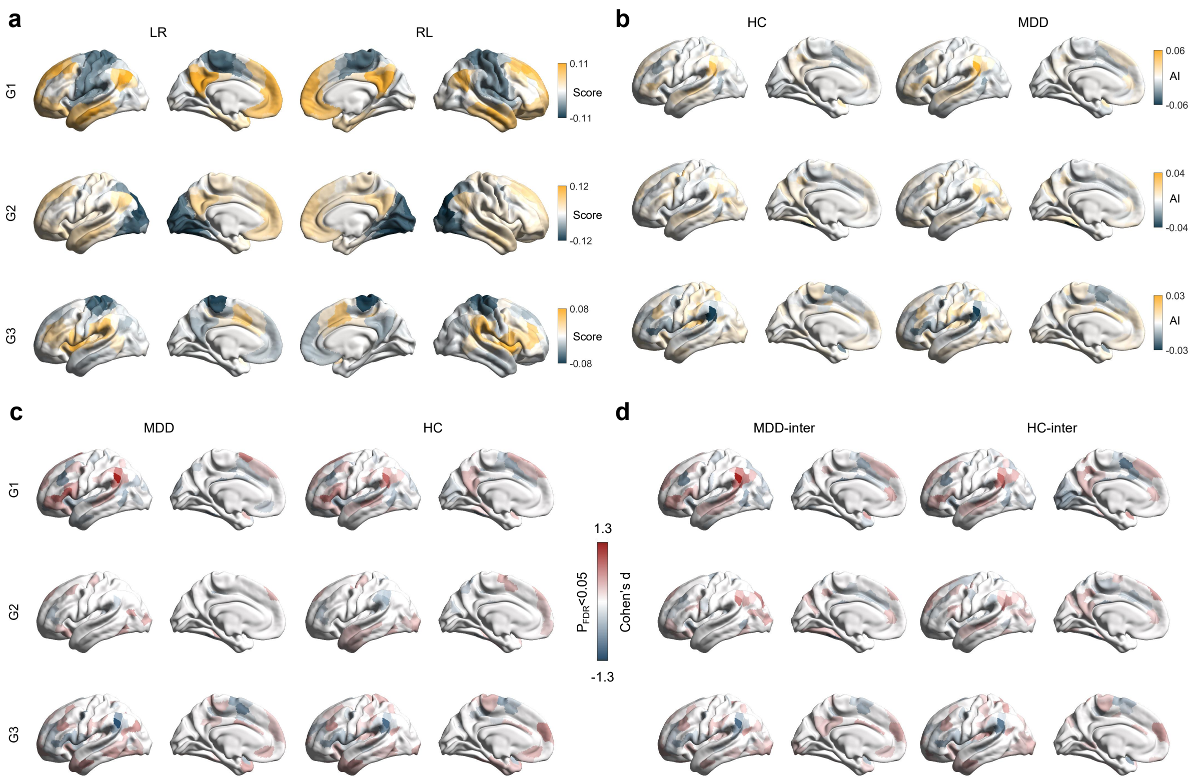

Figure S3. Asymmetry of functional gradients. (**a**) The average inter-hemispheric patterns of the first three gradients across all individuals. LR indicates the left-to-right hemispheric patterns and RL indicates the right-to-left hemispheric patterns. As shown, the LR and RL patterns of functional gradients were highly similar (group-level: r=0.98 for G1, r=0.99 for G2, r=0.97 for G3; individual-level: $r = 0.86 \pm 0.04$ for G1, $r = 0.84 \pm 0.05$ for G2, $r = 0.77 \pm 0.08$ for G3). (**b**) Mean asymmetry scores (AI) of the first three inter-hemispheric gradians (G1, G2, and G3) for MDD and HC groups. (**c**) Cohen's d maps of intra-hemispheric AI for MDD and HC groups. (**d**) Cohen's d maps of inter-hemispheric AI for MDD and HC groups.

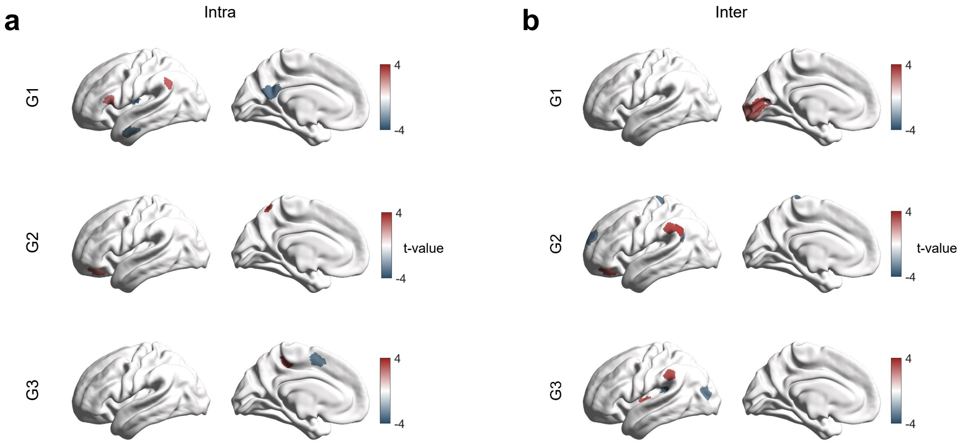

Figure S4. Single-gradient comparisons between the MDD and HC groups. The brain maps of t-values between the MDD and HC groups for intra-hemispheric (**a**) and inter-hemispheric (**b**) gradients.

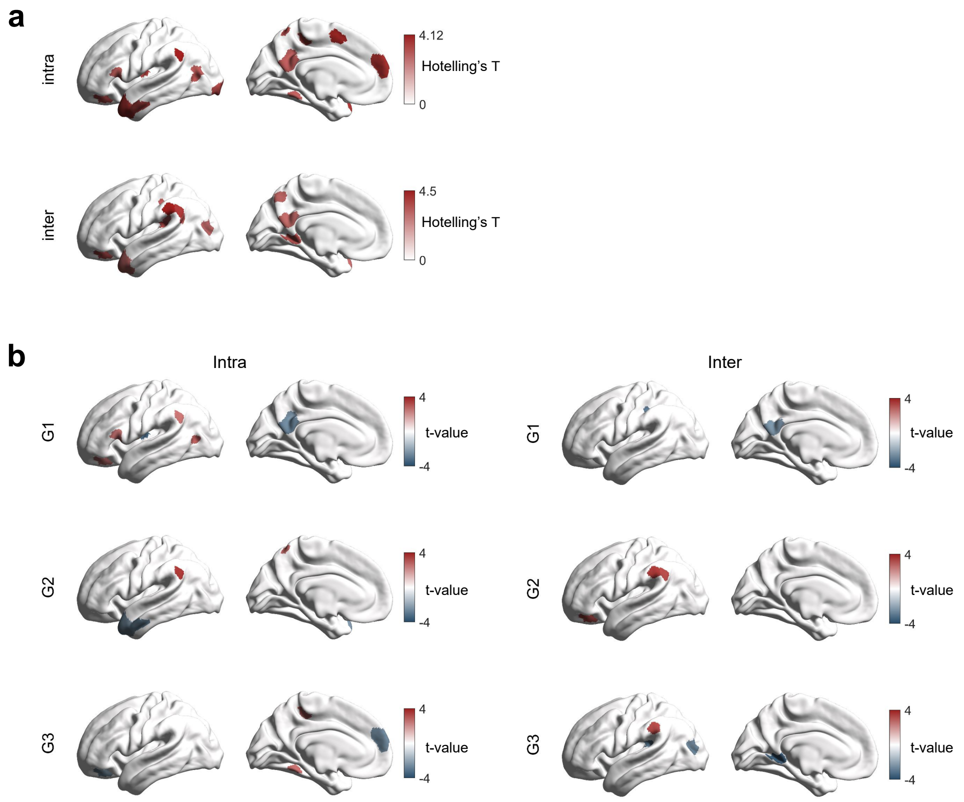

Figure S5. Regional-level MDD-HC comparison using data with GSR. (**a**) Multivariate comparison of intra-hemispheric and inter-hemispheric asymmetry across three gradients (FDR corrected $P < 0.05$). Nodes are colored according to Hotelling's T values. (**b**) Brain maps of t-values between the MDD and HC groups for single-gradient analyses ($P < 0.05/3$).

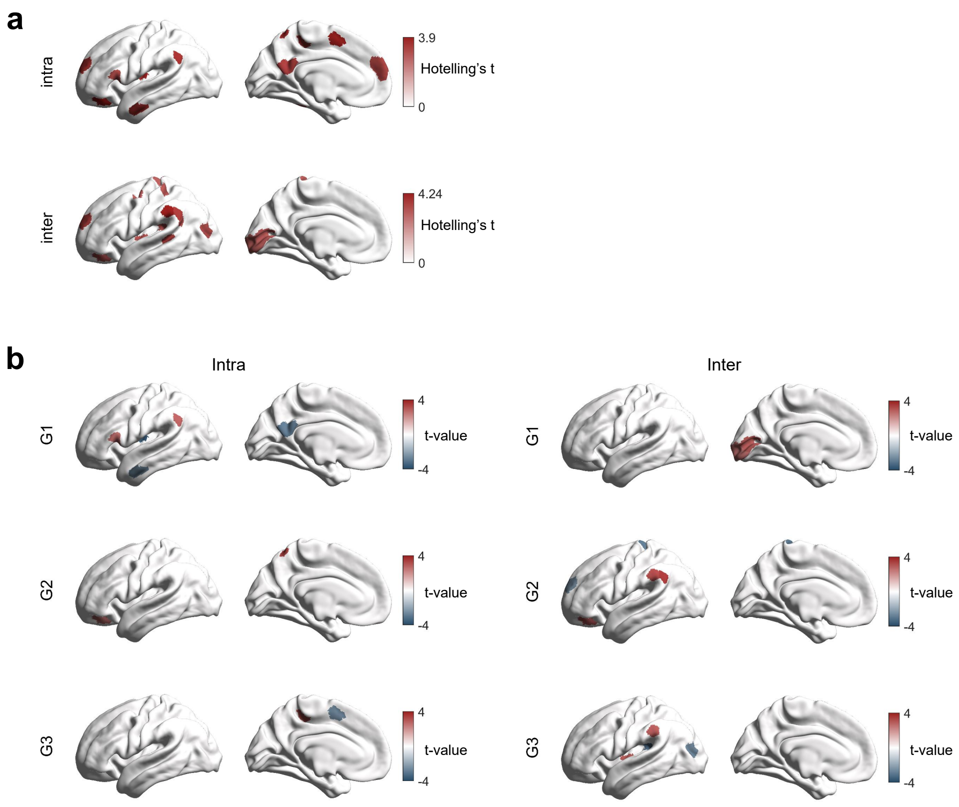

Figure S6. Regional-level MDD-HC comparison using gradient template constructed exclusively from the HC group. (**a**) Multivariate comparison of intra-hemispheric and inter-hemispheric asymmetry across three gradients (FDR corrected $P < 0.05$). Nodes are colored according to Hotelling's T values. (**b**) Brain maps of t-values between the MDD and HC groups for single-gradient analyses ($P < 0.05/3$).

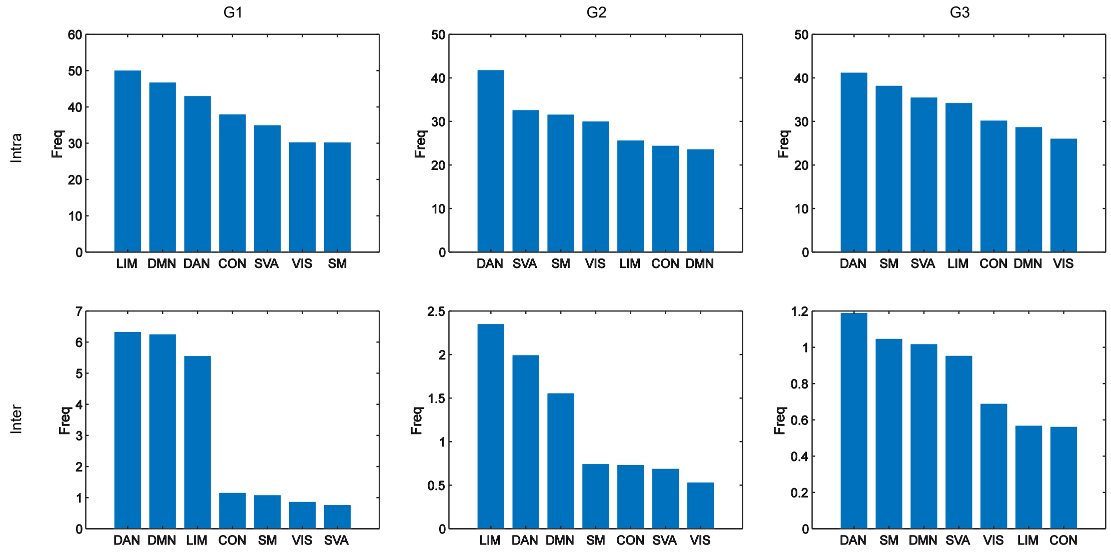

Figure S7. Frequency of selected featured occurred in seven resting-state networks. RSNs: DMN, default mode; CON, control, LIM, limbic, SVA, salience ventral attention, DAN, dorsal attention, SM, somatomotor, and VIS, visual networks.

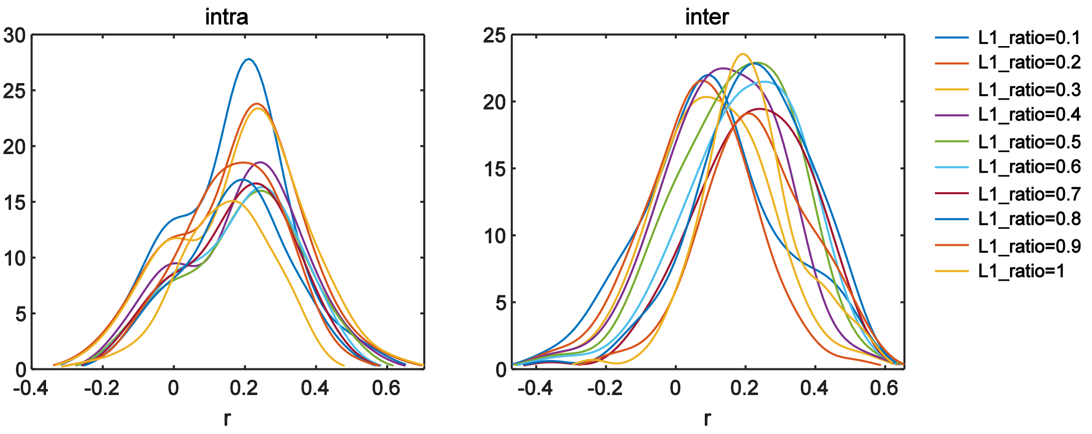

Figure S8. Prediction performance across different L1_ratio parameters (from L1_ratio=0.1 to L1_ratio=1) for intra- and inter-hemispheric features. The performance of elastic net with different L1_ratio parameters was similar. In all cases, the prediction accuracy r significantly exceeded the chance level (10,000 permutations, $P < 0.0001$).
